# A Quality Improvement (Pilot) Project: Psychiatric Medical Education for Foundation Trainees

**DOI:** 10.1192/bjo.2022.123

**Published:** 2022-06-20

**Authors:** Pratibha Nirodi, Imagbe Uwaifo, Christiana Elisha-Aboh, Ogba Onwuchekwa, Rahul Watts, Richard Johnson, Emma Brooks, Lauren Fitzmaurice, Emily Legg, Maggie Robinson, Jess Moncrieff

**Affiliations:** 1 Tees Esk and Wear Valleys NHS Foundation Trust, Harrogate, United Kingdom.; 2 Bradford Teaching Hospitals NHS Foundation Trust, Bradford, United Kingdom; 3 Leeds and York Partnership NHS Foundation Trust, Leeds, United Kingdom; 4 Tees Esk and Wear Valleys NHS Foundation Trust, York, United Kingdom; 5 Sheffield Health and Social Care NHS Foundation Trust, Sheffield, United Kingdom; 6 South West Yorkshire Partnership NHS Foundation Trust, Wakefield, United Kingdom

## Abstract

**Aims:**

Foundation Doctors are exposed to a range of specialties within the Foundation Programme, with 20.9% completing a psychiatry rotation. Those who do not have a psychiatry rotation may have little experience other than what was acquired in undergraduate training, despite being expected to care for patients with mental health problems. According to Mind (2017), one in four people will experience a mental health problem each year thus essential that our medical workforce know and understand the basic principles of psychiatry to aid their management of core psychiatric conditions. The aim of this project was to improve mental health literacy among Foundation Doctors by improving their communication, formulation and risk management skills. Another objective was to encourage uptake to Psychiatry and help plug the high number of unfilled Consultant posts.

**Methods:**

The initial pilot was carried out between January and June 2021 over zoom and the sessions were optional. A survey was completed to find out which topics were most relevant and common themes included MCA/MHA interface, risk management and treatment of various conditions. These themes were incorporated into 90-minute sessions which included interactive case-based discussion in small breakout groups and some didactic teaching. The six session topics were EUPD, Dementia, Depression, Delirium, Substance Misuse and Alcohol Misuse. The sessions were facilitated by clinicians of mixed experience from Foundation Doctors to Consultants. Participant knowledge was tested using pre- and post-session quizzes and a working group reviewed feedback, making relevant changes subsequently.

**Results:**

Feedback was majorly positive, and attendees valued the interactivity, breakout rooms, case studies and choice of topics. Suggested areas of improvement were having more time for discussion, technical difficulties, and less psychiatric ‘jargon’, but these tended to be isolated comments. Five out of six sessions showed an improvement in assessment scores afterwards, with an average improvement of 12.6% (average pre-session score of 70% and average post-session score of 82.6%). One session showed a decrease in the post-session quiz scores which on reflection showed that the questions in the assessment covered material not included in the session.

**Conclusion:**

The virtual programme was an effective way of improving knowledge and confidence in psychiatry. Whilst the sessions were positively received and showed improvements in post-session scores, there were some limitations which will be addressed and used to develop future training. There is now more mental health woven throughout the new Foundation curriculum and expected that much of this content will be covered during Foundation Training.

